# Virtual phacoemulsification surgical simulation using visual guidance and performance parameters as a feasible proficiency assessment tool

**DOI:** 10.1186/s12886-016-0269-2

**Published:** 2016-06-14

**Authors:** Chee Kiang Lam, Kenneth Sundaraj, Mohd Nazri Sulaiman, Fazilawati A. Qamarruddin

**Affiliations:** School of Mechatronic Engineering, Universiti Malaysia Perlis (UniMAP), Kampus Pauh Putra, 02600 Arau, Perlis Malaysia; Faculty of Electronics and Computer Engineering, Universiti Teknikal Malaysia Melaka (UTeM), Hang Tuah Jaya, 76100 Durian Tunggal, Melaka Malaysia; Department of Ophthalmology, Hospital Tuanku Fauziah (HTF), Jalan Kolam, 01000 Kangar, Perlis Malaysia; Department of Ophthalmology, Hospital Tengku Ampuan Rahimah (HTAR), Jalan Langat, 41200 Klang, Selangor Malaysia

**Keywords:** Cataract surgery, Performance assessment, Simulation, Surgical training, Virtual reality

## Abstract

**Background:**

Computer based surgical training is believed to be capable of providing a controlled virtual environment for medical professionals to conduct standardized training or new experimental procedures on virtual human body parts, which are generated and visualised three-dimensionally on a digital display unit. The main objective of this study was to conduct virtual phacoemulsification cataract surgery to compare performance by users with different proficiency on a virtual reality platform equipped with a visual guidance system and a set of performance parameters.

**Methods:**

Ten experienced ophthalmologists and six medical residents were invited to perform the virtual surgery of the four main phacoemulsification cataract surgery procedures – 1) corneal incision (CI), 2) capsulorhexis (C), 3) phacoemulsification (P), and 4) intraocular lens implantation (IOL). Each participant was required to perform the complete phacoemulsification cataract surgery using the simulator for three consecutive trials (a standardized 30-min session). The performance of the participants during the three trials was supported using a visual guidance system and evaluated by referring to a set of parameters that was implemented in the performance evaluation system of the simulator.

**Results:**

Subjects with greater experience obtained significantly higher scores in all four main procedures – CI1 (ρ = 0.038), CI2 (ρ = 0.041), C1 (ρ = 0.032), P2 (ρ = 0.035) and IOL1 (ρ = 0.011). It was also found that experience improved the completion times in all modules – CI4 (ρ = 0.026), C4 (ρ = 0.018), P6 (ρ = 0.028) and IOL4 (ρ = 0.029). Positive correlation was observed between experience and anti-tremor – C2 (ρ = 0.026), P3 (ρ = 0.015), P4 (ρ = 0.042) and IOL2 (ρ = 0.048) and similarly with anti-rupture – CI3 (ρ = 0.013), C3 (ρ = 0.027), P5 (ρ = 0.021) and IOL3 (ρ = 0.041). No significant difference was observed between the groups with regards to P1 (ρ = 0.077).

**Conclusions:**

Statistical analysis of the results obtained from repetitive trials between two groups of users reveal that augmented virtual reality (VR) simulators have the potential and capability to be used as a feasible proficiency assessment tool for the complete four main procedures of phacoemulsification cataract surgery (ρ < 0.05), indicating the construct validity of the modules simulated with augmented visual guidance and assessed through performance parameters.

## Background

Cataract is a general vision illness that is daily diagnosed and reported in a large population of eye patients worldwide. Most of the patients are affected by this illness due to aging [1], diabetes [2], or overexposure to ultraviolet radiation [3].

Traditional master-apprentice surgical training on live patients and wet-lab training on animal cadavers suffer from several drawbacks, which include heterogeneity of anatomic situations, high financial expenditure, limited availability and repeatability, and time constraints. The latest development of virtual surgical simulators allows the performance of a simulation to the level of recreating real-life situations to improve and attain a medical professional's specific competencies [4]. Surgical complications and traumas during an actual operation can be mimicked in a three-dimensional virtual environment to increase the awareness of surgeons and medical residents. Surgical simulators have been developed for various surgical areas, such as endoscopic surgery [5], endovascular surgery [6], and laparoscopic surgery [7]. They have received constructive feedback from numerous clinical validation articles.

EYESi® [8, 9] (VRmagic, Mannheim, Germany) and PhacoVision® [10] (Melerit Medical, Linkoping, Sweden) are the only two commercially available cataract surgery simulators. EYESi® allows for user assessment during the continuous curvilinear capsulorhexis procedure while PhacoVision® can assess users during the phacoemulsification training. The training modules in EYESi® were used to investigate the effect of virtual reality (VR) training in improving the wet-lab performance of capsulorhexis [11]. The results provide objective evidence that training with the EYESi® simulator improves wet-lab operative performance in medical residents with little or no prior microsurgical experience. Recently, [12] conducted a feasibility study on training and assessment using EYESi® and concluded that the system is reliable in differentiating the proficiency level of a user. PhacoVision® on the other hand, had performance indices integrated into its system in order to enhance monitoring and characterize surgical proficiency of users during virtual training of phacoemulsification procedures [13–15]. However, the reported findings on both these simulators were limited to either the capsulorhexis or phacoemulsification procedures. A recent non-commercial simulator [16] simulated and implemented an assessment system to three out the four main procedures in cataract surgery. However, there is no mention of any validation studies by medical professionals for this simulator.

A VR phacoemulsification cataract surgery simulator has been developed in-house and its architecture described in our previous articles [17, 18]. The simulator consists of a personal computer, simulation software, human-computer interface, and a pair of haptic devices to apply and measure force feedback. The main aim of this experimental study was to conduct virtual phacoemulsification cataract surgery to compare performance by users with different proficiency on a virtual reality platform equipped with a visual guidance system and a set of performance parameters. Assessment is done on all four main procedures of phacoemulsification cataract surgery.

## Methods

The proposed study was reviewed and approved by the Malaysian Research Ethics Committee (MREC) and was thus performed in accordance with the principles of the Declaration of Helsinki due to the involvement of human participants. The subjects that participated were briefed of the purpose and objectives of the study and provided their informed written consent followed by completing a questionnaire and duly signing it at the beginning and end of the experiment respectively.

A group of medical professionals from the Department of Ophthalmology at Hospital Tuanku Fauziah, Perlis, Malaysia and Hospital Tengku Ampuan Rahimah, Klang, Malaysia were invited to conduct the validation study of the developed VR simulator for phacoemulsification cataract surgery. The group was composed of six medical residents (Group A) who are undergoing training in Ophthalmology and ten experienced ophthalmologists (Group B). Prior to the actual trial, all of the participants were allowed 30 min of practice time to become familiar with techniques of manoeuvring the stylus of the haptic device and the manipulation of the keyboard and mouse to adjust the view of the virtual surgical environment. A briefing session regarding virtual simulation of phacoemulsification cataract surgery was then given to the participants to highlight the objectives of each of the four main procedure integrated into the simulator – 1) corneal incision (CI), 2) capsulorhexis (C), 3) phacoemulsification (P), and 4) intraocular lens implantation (IOL). Each participant was required to perform the complete virtual phacoemulsification cataract surgery using the simulator for three consecutive trials. The performance of the participants during the three trials was recorded by the simulation system and compiled into an evaluation report for investigation.

A set of parameters, which monitor the training objectives in each procedure of cataract surgery, were selected and implemented into the performance evaluation system of the simulator to assess the performance of the participants. Parallel to this, is the visual guidance system, which sequentially reminds and guides the surgical steps involved in each procedure, to assist the user towards attaining the objectives. The purpose of this dual system is to increase and maintain the awareness and focus of the user when conducting the four main cataract surgery procedures.

### Experimental study platform

**Fig. 1:**
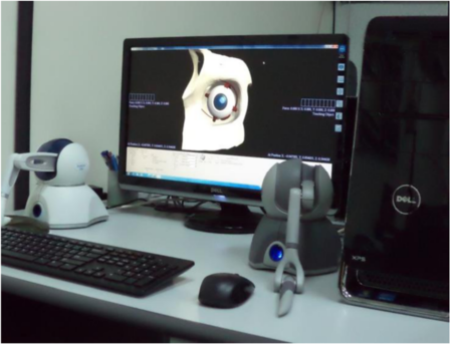
Phacoemulsification Cataract Surgery Training Simulator

The system architecture of the developed simulator consists of three main systems – a simulation system, a haptic rendering system and a graphics rendering system. A pair of SensAble Phantom Omni haptic device is included in the system to provide the control of the virtual surgical instruments during the surgical simulation and to generate force feedback. These devices exhibit six degrees of freedom (DOFs) in position tracking and three DOFs in force feedback. The haptic rendering system is designed to obtain the contact point and force from both of the styli’s movement. The position, orientation, and applied force of the virtual instruments are obtained from the haptic devices' encoders. The information from the stylus is processed by the simulation system to identify the type of surgical activity, such as cutting, grasping, pulling, pushing, sculpting, and emulsifying. The simulation system also processes these data for collision detection and then computes the changes in the geometric data of the eye model to be rendered as dynamic deformations. In addition, the reaction force is also computed and simultaneously fed back to the haptic devices. The graphics rendering system identifies and updates the visual deformation and topological modification on the mesh of the eye model. The changes to the eyeball will be generated as three-dimensional images and output to the users through a computer display. Figure 1 shows the developed phacoemulsification cataract surgery training simulator.

**Fig. 2:**
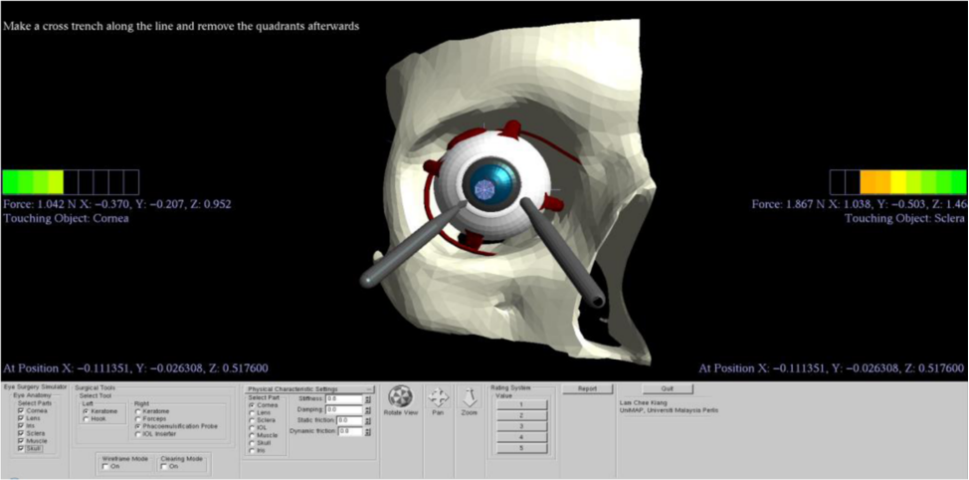
Virtual surgical environment of the simulator

The four main procedures of phacoemulsification cataract surgery, namely corneal incision, capsulorhexis, phacoemulsification, and intraocular lens implantation, were implemented into the simulator as training modules for virtual surgical training and assessment. The visual guidance system, which is capable of providing interactive supervision to the users in each procedure, was designed and incorporated in the developed simulator as a human-computer interface. Figure 2 shows the virtual surgical environment of our simulator.

### Surgical performance parameters and assessment criteria

The proposed set of performance parameters are listed according to the type of procedure, as detailed in Table 1. These surgical parameters were selected and implemented in the developed simulator to evaluate the performance of the user in the main procedures of phacoemulsification cataract surgery through several assessment criteria as listed in Table 2. These parameters and assessment criteria were conceptualized and formulated according to the four modules and required skills through numerous discussions with experienced consultant ophthalmologists.

**Table 1:** Performance parameters for virtual assessment and evaluation

1 – Corneal Incision (CI) Module CI1 – Score for main incision CI2 – Score for side port incision CI3 – Maximum force applied on cornea CI4 – Time taken	
2 – Capsulorhexis (C) Module C1 – Score for capsulorhexis C2 – Number of touches on cornea C3 – Maximum force applied on anterior capsule C4 – Time taken	
3 – Phacoemulsification (P) Module P1 – Percentage of cataract fragments removed P2 – Score for divide and conquer P3 – Number of touches on posterior capsule P4 – Number of touches on iris P5 – Maximum force applied on posterior capsule P6 – Time taken	
4 – IOL Implantation (IOL) Module IOL1 – Score for implantation IOL2 – Number of touches on posterior capsule IOL3 – Maximum force applied on posterior capsule IOL4 – Time taken

**Table 2:** Assessment criteria of performance parameters

Score	Training Objectives					
	Prevention of the damage of the structure				Precision of the restoration of the vision	
	Performance Parameters Measured					
	Corneal Incision		Capsulorhexis	Phacoemulsification		IOL Implantation
	Main Incision	Side Port		Divide & Conquer	Cataract Extraction	
	Distance away from ideal position (Fig. 3)		Distance away from ideal circular path (Fig. 4)	Distance away from ideal cross-trench (Fig. 5)	Percentage of cataract fragments removed (Fig. 5)	Distance away from ideal position (Fig. 6)
10	<2 %	<2 %	<2 %	<2 %	>90 %	<2 %
9	<4 %	<4 %	<4 %	<4 %	>80 %	<4 %
8	<6 %	<6 %	<6 %	<6 %	>70 %	<6 %
7	<8 %	<8 %	<8 %	<8 %	>60 %	<8 %
6	<10 %	<10 %	<10 %	<10 %	>50 %	<10 %
5	<12 %	<12 %	<12 %	<12 %	>40 %	<12 %
4	<14 %	<14 %	<14 %	<14 %	>30 %	<14 %
3	<16 %	<16 %	<16 %	<16 %	>20 %	<16 %
2	<18 %	<18 %	<18 %	<18 %	>10 %	<18 %
1	>18 %	>18 %	>18 %	>18 %	<10 %	>18 %

### Evaluation of procedures using performance parameters and visual guidance system

Phacoemulsification cataract surgery usually begins by making a main incision and side port incision at the temporal limbus of the cornea to allow the insertion of surgical instruments. The anterior surface of the capsule that encloses the nucleus is the removed and this procedure is called capsulorhexis. The operation is then continued by using a phaco-probe, whose tip vibrates at an ultrasonic frequency to divide the crystallised lens into four identical quadrants and emulsify the cataract into small pieces, while the aspiration port at the tip of the phaco-probe removes the fragments by suction from a pump. A foldable IOL is finally implanted into the capsular bag at the end of the cataract surgery to restore the vision of the patient.

#### Corneal incision module

**Fig. 3:**
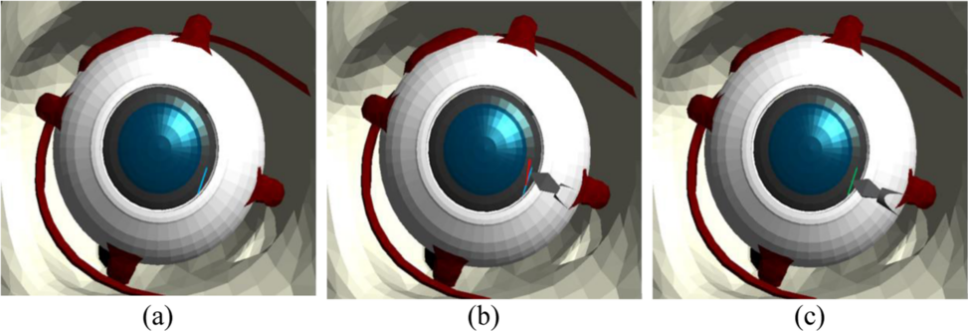
Visual guidance for corneal incision – **a** ideal, **b** bad and **c** precise incision

The position and size of the tunnel are the main concerns during corneal incision because these influence the possibility of astigmatism and provide indication if the created wound from the incision is free from sutures. The embedded visual guidance system provides informative guide on top of the display unit to help the user perform the corneal incision properly. This system renders a line at the ideal position on the temporal limbus of the cornea as a guide for the user to make the incision as illustrated in Fig. 3.

Assessment in this module is conducted by computing the difference in the distance between the ideal position of the incision and that made by the user. We also recorded the maximum force applied on the surface of the cornea and the time taken to complete the corneal incision procedure.

#### Capsulorhexis module

**Fig. 4:**
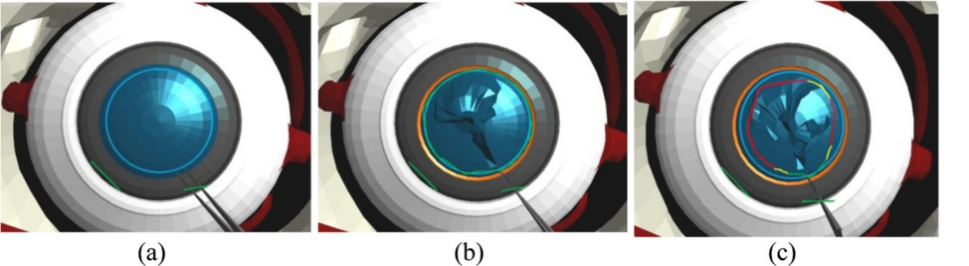
Visual guidance for capsulorhexis – **a** ideal, **b** accurate and **c** least-accurate trajectories

One of the main challenges associated with the capsulorhexis module is that the flap created by the forceps should not be torn outward because it will lead to the damage of the capsular bag, which is the ideal place for the insertion of the IOL. In addition, the capsular bag also acts as the barrier between the anterior and posterior chambers. To aid the user through this procedure, the visual guidance system indicates the correct path for grasping and tearing the surface of the capsule by rendering a trajectory in the form of a circle at the centre of the anterior capsule as illustrated in Fig. 4.

The ideal outcome of the capsulorhexis module is a circular opening with a standard radius centred on the lens of the eye. This is evaluated by computing the difference in the distance of the circular path from the centre of the lens. This provides an indication of the accuracy of the grasping and tearing motion performed on the anterior capsule. Similar to the previous module, we also recorded the maximum force applied on the anterior capsule, the number of touches on the cornea during the capsulorhexis operation and the time taken to complete the procedure.

#### Phacoemulsification module

**Fig. 5:**
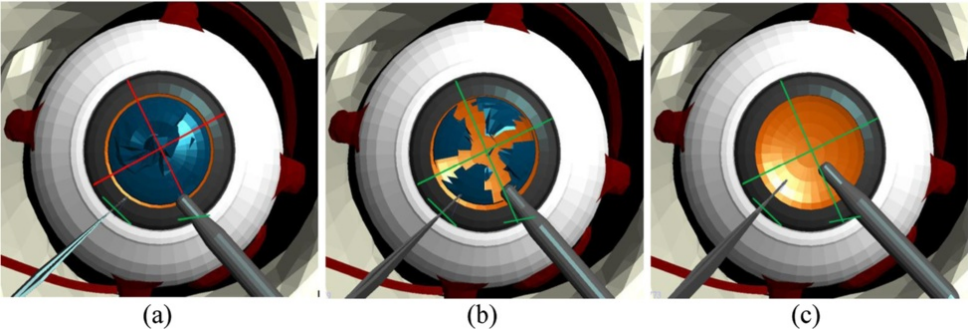
Visual guidance for phacoemulsification – **a** cross trench indicator, **b** divide and conquer and **c** ideal removal of cataract

The main objective of the phacoemulsification module is the complete removal of the cataract from the capsular bag without damaging the posterior capsule. The posterior capsule must be retained intact to later provide support for the IOL. This capsule also acts as the barrier between the anterior and posterior chambers. To guide the user towards applying an efficient technique to remove the cataract, the visual guidance system renders a cross on the ideal position of incision on the cornea for the user to make a trench across the centre of the nucleus as shown in Fig. 5.

This module is evaluated by assessing the ability of the user to divide the cornea into four equivalent quadrants and the ability to remove all cataract fragments in each quadrant from the capsular bag. We also record the number of touches and the maximum force applied on the posterior capsule to increase the awareness of the user to the effects of damaging the cornea, which may cause the rupture of the capsular bag leading to vitreous haemorrhage and retinal detachment. Additional assessment criteria include the number of touches on the cornea, the number of touches on the iris, and the time taken to complete the phacoemulsification module.

#### IOL implantation module

**Fig. 6:**
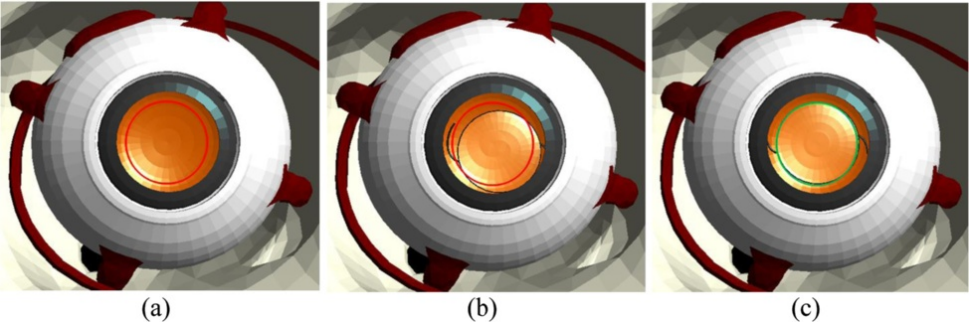
Visual guidance for IOL implantation – **a** ideal, **b** bad and **c** precise implantation

The main purpose of the IOL implantation module is the precise positioning of the artificial lens inside the capsular bag. The lens must be inserted at the centre of the capsular bag such that the light that penetrates into the eye can be focused onto the retina. To guide the user towards this objective, the visual guidance system renders a circle centred inside the capsular bag as illustrated in Fig. 6 to indicate the correct position for the IOL implantation.

Accordingly, the performance of the user during the IOL implantation procedure is evaluated by comparing the distance between the ideal position of the IOL and the position that is finally attained by the user. As in the previous modules, we also record the number of touches on the posterior capsule, the maximum force applied on the surface of the posterior capsule, and the time taken to complete the module.

## Results

A total of 16 subjects participated in the assessment of virtual phacoemulsification cataract surgery to compare performance by users with different proficiency on a VR platform equipped with a visual guidance system and a set of performance parameters. The subjects were separated into two groups – Group A was composed of medical trainees and Group B was composed of experienced ophthalmologists. Table 3 presents the descriptive statistics of the subjects according to their number of years of training in ophthalmology and the number of cataract surgical operations conducted. Each subject completed three trials of VR training session successfully and their surgical performance was recorded in the simulation system and compiled into an evaluation report. Statistical analysis was the conducted using the SPSS Version 20 (IBM Corp., USA) statistics software using the score and recorded values derived from the surgical performance parameters and assessment criteria as the basis of measurement.

**Table 3:** Descriptive statistics of the subjects

Characteristic	Group A	Group B
Number	6	10
Sex, N (%)		
Female	4 (66.7)	8 (80.0)
Male	2 (33.3)	2 (20.0)
Age, Y		
Mean	27.8	41.2
Range	26 – 31	34 – 57
Level of training		
Postgraduate students	6	–
Staff	–	10
Cataract surgeries performed		
Mean	11.7	700.3
Range	10 – 20	100 – 2000
Years of practice		
Mean	3.2	13.4
Range	2 – 4	10 – 20

The recorded data of each subject per group per trial session per performance parameter was used for the analysis. For both Group A and B, inspection of the results using the Shapiro-Wilk normality test revealed that the scores were not normally distributed for all performance parameters. Therefore, a Mann–Whitney test was run on the data. Tables 4, 5, 6, and 7 present the results.

**Table 4:** Performance between groups on the Corneal Incision (CI) module

Performance parameter	Mean (SD)		Z statistic	ρ – value
	Group A (*n* = 6)	Group B (*n* = 10)		
CI1	7.9 (1.18)	9.2 (0.55)	−4.272	0.038*
CI2	8.2 (1.10)	9.1 (0.48)	−3.516	0.041*
CI3	0.9 (0.40)	0.6 (0.07)	−4.506	0.013*
CI4	151.6 (31.95)	109.6 (7.96)	−4.517	0.026*

Note: *Statistically Significant using Mann–Whitney test (ρ < 0.05)

**Table 5:** Performance between groups on the Capsulorhexis (C) module

Performance Parameter	Mean (SD)		Z statistic	ρ – value
	Group A (*n* = 6)	Group B (*n* = 10)		
C1	7.1 (1.23)	8.9 (0.33)	−5.302	0.032*
C2	15.1 (7.19)	5.8 (1.30)	−4.851	0.015*
C3	0.9 (0.29)	0.5 (0.08)	−4.818	0.027*
C4	296.6 (37.63)	269.8 (12.90)	−2.375	0.018*

Note: *Statistically Significant using Mann–Whitney test (ρ < 0.05)

**Table 6:** Performance between groups on the Phacoemulsification (P) module

Performance Parameter	Mean (SD)		Z statistic	ρ – value
	Group A (*n* = 6)	Group B (*n* = 10)		
P1	8.2 (0.85)	9.4 (0.14)	−0.617	0.077*
P2	8.1 (1.07)	9.3 (0.99)	−4.934	0.035*
P3	14.0 (5.06)	7.0 (1.25)	−4.904	0.015*
P4	5.8 (2.13)	4.2 (0.85)	−3.047	0.042*
P5	1.0 (0.49)	0.7 (0.05)	−3.297	0.021*
P6	549.6 (35.06)	436.2 (14.25)	−5.751	0.028*

Note: *Statistically Significant using Mann–Whitney test (ρ < 0.05)

**Table 7:** Performance between groups on the Intraocular Lens implantation (IOL) module

Performance Parameter	Mean (SD)		Z statistic	ρ – value
	Group A (*n* = 6)	Group B (*n* = 10)		
IOL1	8.3 (1.12)	9.4 (0.50)	−3.628	0.011*
IOL2	4.4 (2.87)	1.8 (0.81)	−3.736	0.048*
IOL3	1.0 (0.57)	0.4 (0.12)	−4.266	0.041*
IOL4	167.8 (42.00)	103.7 (14.14)	−4.836	0.029*

Note: *Statistically Significant using Mann–Whitney test (ρ < 0.05)

In all the four modules of the simulator, subjects in Group B faired better in all the performance parameters compared with subjects in Group A (except P1). Those with greater experience performed better in all the scored components of the modules – CI1 (ρ = 0.038), CI2 (ρ = 0.041), C1 (ρ = 0.032), P2 (ρ = 0.035) and IOL1 (ρ = 0.011). Similarly, experience proved to be pivotal in determining the duration to complete all four modules – CI4 (ρ = 0.026), C4 (ρ = 0.018), P6 (ρ = 0.028) and IOL4 (ρ = 0.029). Positive correlation was observed between experience and the ability to be precise and accurate as observed in the anti-tremor performance parameters – C2 (ρ = 0.026), P3 (ρ = 0.015), P4 (ρ = 0.042) and IOL2 (ρ = 0.048). Group B also performed better in all the anti-rupture performance parameters indicating positive correlation between experience and the ability to control and exert – CI3 (ρ = 0.013), C3 (ρ = 0.027), P5 (ρ = 0.021) and IOL3 (ρ = 0.041). However, in our simulator, experience did not produce any significant difference between the groups with regards to P1 (ρ = 0.077). These statistical results indicate that proficiency, largely acquired through experience, can be objectively observed, assessed and distinguished in the four main modules presented in this work.

## Discussion

The idea of using a virtual guidance system coupled with performance parameters in a virtual surgical simulator is not entirely new. Our findings reveal three other VR simulators in the literature [8–10, 16], of which two have been commercialized at some point in the early development stages. To the best of our knowledge, none of these simulators simulate completely all four main procedures of the cataract surgery. These simulators have all implemented to some extent a virtual guidance system. They however differ in the breadth and depth of assessment. As mentioned earlier, the performance index or parameters of the simulators in [8–10] were limited to either the capsulorhexis or the phacoemulsification procedures only. The final simulator by [16] extended the application of performance parameters to three out of four main procedures. To this effect, we have extended the breadth of assessment in our simulator to the entire four main procedures and presented the results.

The depth of assessment, reflected in the number of performance parameters, during the four main procedures can vary among ophthalmologists, depending on which procedure and skills they deem critical from experience. To this effect, we conducted a broad similarity test between our proposed performance parameters and the previously existing ones. The results indicate that for the first three procedures, we covered all previously assessed critical areas. In fact, we even proposed an additional performance parameter for the side port incision in the corneal incision module (previously [16] only simulated and assessed the main incision). We have further proposed the addition of four performance parameters to assess the IOL implantation, which was not assessed previously. These additions extend the depth of assessment in our simulator to cover all four main procedures of phacoemulsification cataract surgery.

The statistical results from the validation study, obtained between groups indicate that it is possible to assess, evaluate and distinguish proficiency in the surgical skills of phacoemulsification cataract surgery based on virtual performance using the four proposed assessment modules. The results (% of improvement of the performance parameter) reveal that experienced ophthalmologists (Group B) performed better (total score obtained) – CI1 (14 %), CI2 (10 %), C1 (20 %), P2 (13 %) and IOL1 (12 %) and were more efficient (total time taken) – CI4 (38 %), C4 (10 %), P6 (26 %) and IOL4 (62 %) in all four assessment modules. Anti-tremor (# of touches) assessment also improved with greater experience – C2 (160 %), P3 (100 %), P4 (38 %) and IOL2 (144 %). Anti-rupture (maximum applied force) assessments also showed positive outcome for subjects in Group B – CI3 (50 %), C3 (80 %), P5 (43 %) and IOL3 (150 %). Although P1 was not observed to be significantly different, an improvement of 13 % was nevertheless observed between groups.

Our results concur with previously obtained findings. The simulator developed in [10, 13] incorporated performance indices to characterize the performance of users in the phacoemulsification (P) procedure. The evaluation of these performance indices were conducted separately on two different groups, naives [15] and experts [14]. The statistical results indicated that performance varies among groups with different levels of experience. Another statistical analysis in [12] was conducted using the simulator developed in [8, 9] on the capsulorhexis (C) procedure. The subjects were distributed into four groups with different levels of experience in ophthalmology. The statistical results in two of the three assessment modules of the capsulorhexis (C) procedure showed that the group with greater experience achieved significantly better results than groups with lesser experience. These studies together with ours further establish that virtual ophthalmic surgical simulators have great potential to be used as a feasible alternative platform for proficiency assessment.

This work nevertheless has its limitation. The findings have yet to be compared with the traditional master-apprentice and wet-lab training to ascertain the approach using VR simulators in order for it to be introduced in the current curriculum of phacoemulsification cataract surgery. An experimental study similar to the one conducted in [11] has to be conducted. The study will involve a novice group subjected to VR training and a control novice group which would not undergo VR training. Subsequently, a wet-lab assessment will then be conducted, and their performances compared to determine whether the group that underwent VR training performed better.

## Conclusions

The application of VR simulators is not an entirely new technology but its application in the area of phacoemulsification cataract surgery for the purpose of virtual surgical training and assessment is still in its infancy stage. The results of the experimental study presented in this work demonstrate that VR simulators have the potential and capability to be applied as a feasible proficiency assessment tool on the complete four main procedures of phacoemulsification cataract surgery. The statistical analyses of the conducted assessment based on repetitive trials between two groups of users, through the application of a visual guidance system and a set of performance parameters, indicate 1) the construct validity of the modules simulated with augmented visual guidance, 2) proficiency can be objectively assessed using the proposed performance parameters and 3) proficiency can be classified or distinguished using the modules (ρ < 0.05).
